# Inhibition of Neuronal Apoptosis and Axonal Regression Ameliorates Sympathetic Atrophy and Hemodynamic Alterations in Portal Hypertensive Rats

**DOI:** 10.1371/journal.pone.0084374

**Published:** 2014-01-06

**Authors:** Nahia Ezkurdia, Imma Raurell, Sarai Rodríguez, Antonio González, Rafael Esteban, Joan Genescà, María Martell

**Affiliations:** 1 Hepatic Diseases Laboratory, Liver Unit-Department of Internal Medicine, Hospital Universitari Vall d'Hebron, Vall d'Hebron Institut de Recerca (VHIR), Universitat Autònoma de Barcelona, Barcelona, Spain; 2 Centro de Investigación Biomédica en Red de Enfermedades Hepáticas y Digestivas (CIBERehd), Instituto de Salud Carlos III, Madrid, Spain; Bambino Gesù Children Hospital, Italy

## Abstract

**Background and Aim:**

A neuronal pathway participates in the development of portal hypertension: blockade of afferent sensory nerves in portal vein ligated (PVL) rats simultaneously prevents brain cardiovascular regularory nuclei activation, neuromodulator overexpression in superior mesenteric ganglia, sympathetic atrophy of mesenteric innervation and hemodynamic alterations. Here we investigated in PVL rats alterations in neuromodulators and signaling pathways leading to axonal regression or apoptosis in the superior mesenteric ganglia and tested the effects of the stimulation of neuronal proliferation/survival by using a tyrosine kinase receptor A agonist, gambogic amide.

**Results:**

The neuronal pathway was confirmed by an increased neuronal afferent activity at the vagal nodose ganglia and the presence of semaphorin3A in sympathetic pre-ganglionic neurons at the intermediolateral nucleus of the spinal cord of PVL rats. Expression of the active form of tyrosine kinase receptor A (phosphorylated), leading to proliferation and survival signaling, showed a significant reduction in PVL comparing to sham rats. In contrast, the apoptotic and axonal retraction pathways were stimulated in PVL, demonstrated by a significant overexpression of semaphorin 3A and its receptor neuropilin1, together with increases of cleaved caspase7, inactive poly(ADP-ribose) polymerase and Rho kinase expression. Finally, the administration of gambogic amide in PVL rats showed an amelioration of hemodynamic alterations and sympathetic atrophy, through the activation of survival pathways together with the inhibition of apoptotic cascades and Rho kinase mediated axonal regression.

**Conclusion:**

The adrenergic alteration and sympathetic atrophy in mesenteric vessels during portal hypertension is caused by alterations on neuromodulation leading to post-ganglionic sympathetic regression and apoptosis and contributing to splanchnic vasodilation.

## Introduction

The hemodynamic alteration of portal hypertension in patients with liver cirrhosis contributes to most of the clinical manifestations of the disease: gastrointestinal bleeding, hepatic encephalopathy, ascites and renal failure. In the physiopathology of this vascular alteration, splanchnic vasodilation plays an essential role in initiating the hemodynamic process. Regardless of the extensively demonstrated humoral factors involved in splanchnic vasodilatation [1], [2], we have previously shown a remarkable local alteration of the mesenteric adrenergic system (inhibition of adrenergic genes and proteins and atrophy of sympathetic structures), as a possible contributory factor to splanchnic arterial vasodilation [3], [4]. It has also been proposed that during portal hypertension, pressure changes detected at portal level could be the afferent sensory signal triggering this hemodynamic response [5]. We have demonstrated that blockade of sensory afferent nerves in portal hypertensive rats, simultaneously prevents c-fos activation in cardiovascular regulatory nuclei of the brain, hemodynamic alterations and sympathetic atrophy of nerves innervating the superior mesenteric artery (SMA) [6]–[8].

**Figure 1:**
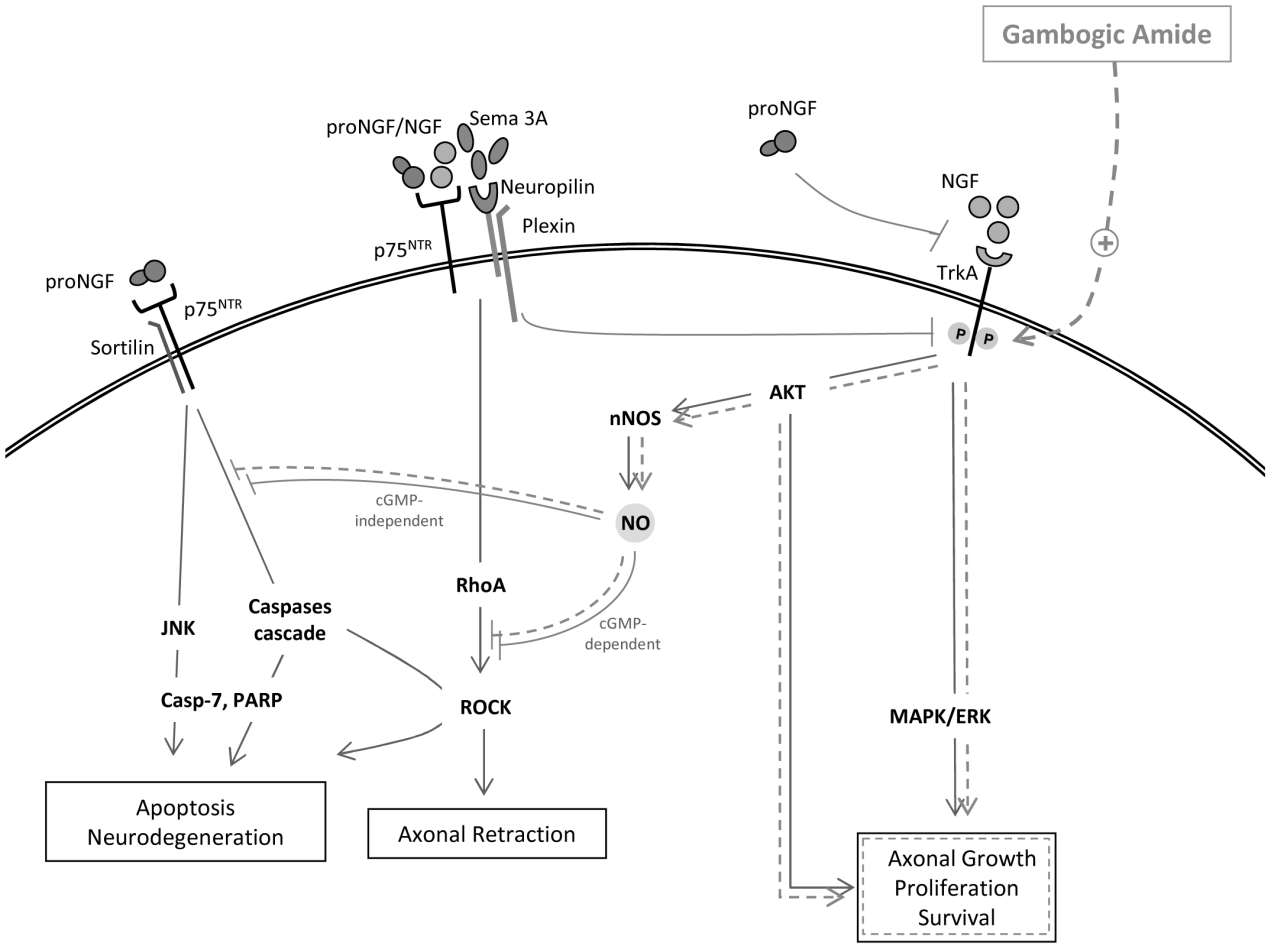
Interplaying signaling pathways responsible for the modulation of axonal survival and death. NGF, nerve growth factor; proNGF, NGF precursor; TrkA, tyrosine kinase receptor A, p75NTR, neurotrophin receptor p75; Sema3A, semaphorin 3A; JNK, c-Jun N-terminal kinase; RhoA, Ras homolog family member A; ROCK, Rho kinase; MAPK/ERK, mitogen activated protein kinase; AKT, protein kinase B; nNOS, neuronal nitric oxide synthase; NO, nitric oxide; Casp-7, cleaved caspase 7; PARP, poly(ADP-ribose) polymerase.

Considering this neuronal pathway, the signal responsible for the post-ganglionic sympathetic atrophy could be originated in pre-ganglionic fibers that synapse at the superior mesenteric ganglion (SMG). By analyzing the SMG of portal vein ligated (PVL) rats, we have shown an overexpression of nerve growth factor (NGF) and its precursor (proNGF), as well as an increase in the neurotrophin receptor p75 (p75NTR) and in the chemorepellent molecule semaphorine 3A (Sema3A) [6]. This altered neuromodulator expression in the SMG of PVL rats suggested that the adrenergic downregulation and sympathetic atrophy observed in mesenteric vessels might have their origin in disorders leading to axonal regression and/or apoptosis. Interplaying signaling pathways are responsible for the modulation of axonal survival and death (figure 1). While NGF is a neurotrophin responsible for neuronal survival and growth, its precursor pro-NGF can directly induce neuronal regression/death, when its effect is mediated by p75^NTR^ [9], [10] or indirectly through the inhibition of tyrosine kinase receptor A (TrkA) NGF-mediated activation [11]. Sema3A strongly inhibits axonal growth in situations of intense NGF stimulation, by dephosphorylating the NGF main neuronal receptor TrkA [12]. In turn, when active, TrkA promotes neuronal survival through the silencing of the apoptotic signal mediated by p75^NTR^ [13]. Finally, neurotrophin binding to p75^NTR^ is necessary for Sema3A-mediated axonal regression/growth cone collapse [14], [15].

This study was aimed to (i) confirm the implication of the neuronal pathway in portal hypertension by analyzing two intermediate structures: the nodose ganglia in the afferent direction, containing the neuronal soma of afferent sensitive nerves running along the vagus nerve and the intermediolateral nucleus of the spinal cord in the efferent direction, containing the neuronal bodies of pre-ganglionic sympathetic nerves; (ii) further explore the neuromodulators and signaling pathways that, in the SMG of PVL rats, lead to Sema3A/ProNGF/p75^NTR^-mediated axonal regression or apoptosis; and (iii) find out whether treatment of PVL rats with gambogic amide, a TrkA agonist, inhibits this neurodegenerative pathway, and consequently, the hemodynamic alteration and sympathetic atrophy of portal hypertension disappear or improve.

## Materials And Methods

### Ethic statement

Animals received humane care and all efforts were made to minimize suffering, according to the recommendations of the European Commission on the protection of animals used for experimental and scientific purposes (http://ec.europa.eu/environment/chemicals/labanimals/pdf/report_ewg.pdf). All animal experiments were approved by The Animal Care Committee of the Vall d'Hebron Institut de Recerca (VHIR), Barcelona, Spain (Associate permit number:5953).

### Experimental portal hypertension

Prehepatic portal hypertension was induced by partial portal vein ligation as described previously [3]. Two groups of 7 portal hypertensive rats and 7 sham operated rats were studied. A third group of 16 PVL animals were treated daily with gambogic amide (n = 8) (Enzo, Farmingdale, NY, USA) or vehicle (1% DMSO) (n = 8), beginning the same day of the PVL surgery and during 14 days. Gambogic amide was intreperitoneally (i.p.) injected at 0.35 mg/kg body weight (BW). In order to have normal state reference values, 8 sham rats were also performed.

### Hemodynamic measurements

Two weeks after PVL surgery or sham operation, animals were anaesthetized with a mix of ketamine hydrochloride (100 mg/kg BW, i.p.) plus midazolam (5 mg/kg BW, i.p.) for continuous measurement of mean arterial pressure (MAP, mmHg), portal pressure (PP, mmHg) and SMA blood flow (SMABF, ml/min.100 g). SMA resistance (SMAR, mmHg/ml.min.100 g) was calculated as (MAP-PP)/SMABF.

### Sample extraction

See data S1 for details.

### Antibodies for Western blot analysis

Protein expression analysis was performed in nodose ganglion samples for calcitonin gene related protein (CGRP) (diluted 1/500) (Biomol, Pennsylvania PA, USA) and in SMG for tyrosine hydroxylase (Th) (dil 1/600), Sema 3A, sortilin (Sort1) (diluted 1/1000) (Abcam, Cambridge, UK), TrkA, Rho kinase (ROCK) (diluted 1/200) (Santa Cruz Biotechnology, Santa Cruz, CA, USA), phospho-TrkA (pTrkA), mitogen activated protein kinase (MAPK), phospho-MAPK (pMAPK), c-Jun N-terminal kinase (JNK), phospho-JNK (pJNK), protein kinase B (AKT), phospho-AKT (pAKT), cleaved caspase7 (casp-7), poly(ADP-ribose) polymerase (PARP) (diluted 1/1000) (Cell Signaling Technology, Beverly, MA, USA) and neural nitric oxide synthase (nNOS) (1/1000) (BD Biosciences, San Jose, CA, USA). See data S1 for details.

### Immunofluorescence

Serial 8 µm sections of spinal cord were analyzed for vesicular acetylcholine transporter (VAChT, diluted 1∶200) (Novus Biologicals, Littleton, CO, USA) immunoreactivity, as a marker of preganglionic sympathetic cholinergic neurons. Successive slides were used to analyze Sema3A immunoreactivity(diluted 1∶200). Bound antibodies were incubated with anti-goat IgG DyLight 594 (diluted 1∶500) (Bethyl, Montgomery, TX, USA) for VAChT and with anti-rabbit-FITC (diluted 1/500) (Abcam, Cambridge, UK) for Sema3A

Paraffin-embedded sections of SMG were incubated with primary antibodies neuropilin-1 (Nrp-1) (R&D Systems, Minneapolis, MN, USA) (diluted 1∶20), p75^NTR^ (diluted 1/500) (Abcam), Sort-1 (diluted 1/200) and TrkA (diluted 1/200). Bound antibodies were incubated with anti-rabbit-FITC. See data S1 for details.

### Immunohistochemistry

Total nervous area and Th staining area was carried out by immunohistochemistry on paraffin-embedded sections of SMA incubated with primary antibody anti-Th (diluted 1∶250) as described [4], [8].

### Statistical analysis

Normally distributed values were compared using Student's t-test and expressed as mean ± S.E.M. Statistical significance was established at p<0.05.

## Results

### The afferent signal through the vagus nerve is increased in portal hypertension

**Figure 2:**
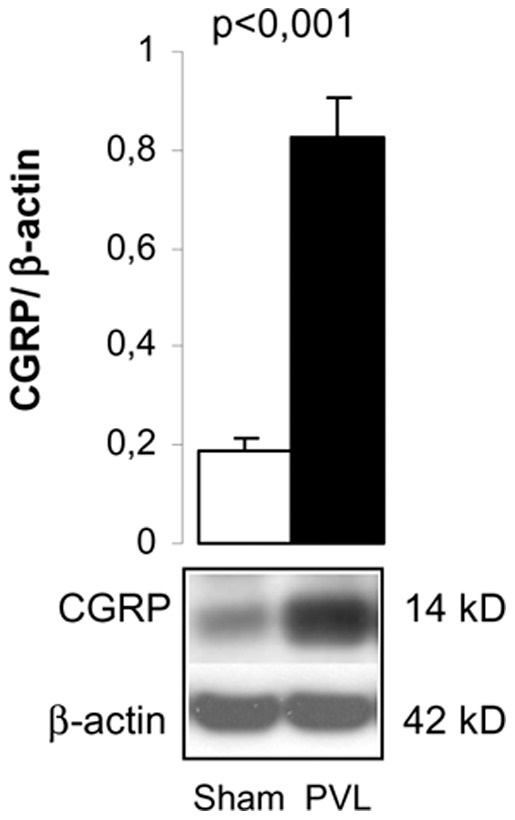
Calcitonin gene related protein (CGRP) in the nodose ganglion. Bar diagram showing CGRP quantitation by Western blot analysis in sham (n = 6) and PVL (n = 7). Representative Western blot is shown below.

Visceral afferent sensitive nerves release the neurotransmitter CGRP upon stimulation, not only from the terminals of primary afferent fibers, but also from the cell bodies and along axons. CGRP expression was analyzed in the nodose ganglia containing the neuronal soma of afferent sensitive nerves running along the vagus nerve. The result clearly showed a significant 4-fold increase of CGRP expression in PVL rats compared to sham (p<0.001) (figure 2).

### Expression of semaphorin 3A in pre-ganglionic cholinergic neurons

**Figure 3:**
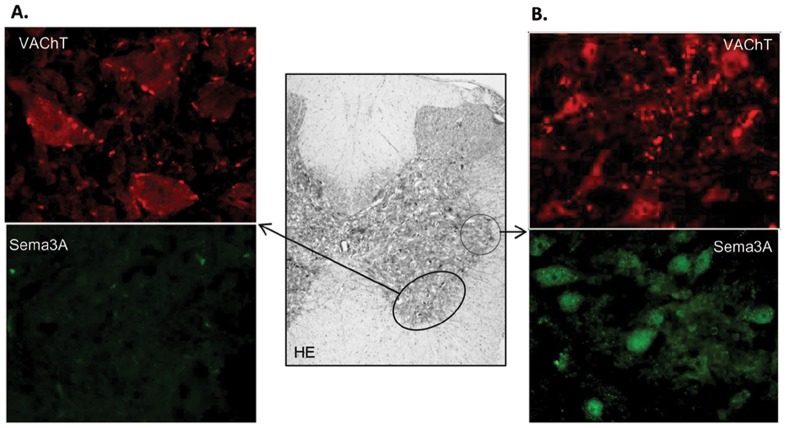
Semaphorin 3A (Sema3A) in the intermedioratelal nucleus of the spinal cord in PVL rats. (A) Magnifications (400×) of the ventral horn area of the spinal cord (middle gray color image at 4×), containing cholinergic neurons, showing strong VAChT immunofluorescent signal and null immunoreactivity for Sema3A. (B) Magnifications (400×) of the spinal cord intermediolateral nucleus containing cholinergic preganglionic sympathetic neurons. These neurons show positive immunofluorescent signal for both VAChT and Sema3A. HE, hematoxilin-eosin staining.

In a previous study, Sema3A overexpression was localized at the cholinergic axon fibers surrounding adrenergic neurons in the SMG (8). We hypothesized a cholinergic pre-ganglionic origin for this increase in Sema3A and searched for its presence in the intermediolateral nucleus of the spinal cord (spinal segments from T9 to T13) containing the cell bodies of preganglionic sympathetic neurons that synapse at the SMG. As shown in figure 3, cholinergic VAChT inmunoreactive neurons were present in the ventral horn (figure 3A) and in the intermediolateral nucleus (figure 3B) of the spinal cord. As expected, the size and staining pattern were different between neurons from both locations, being smaller and uniformly stained neurons at the intermediolateral nucleus, without the punctuated appearance of the cholinergic neurons at the ventral horn. In successive sections, positive Sema3A immunofluorescent signal was only found in neurons located at the intermediolateral nucleus of the spinal cord of PVL rats, showing a similar size and staining pattern as cholinergic neurons (figure 3B).

### Tyrosine hidroxylase deficit and semaphorine 3A/neuropilin 1 overexpression in adrenergic neurons of the superior mesenteric ganglion in PVL rats

**Figure 4:**
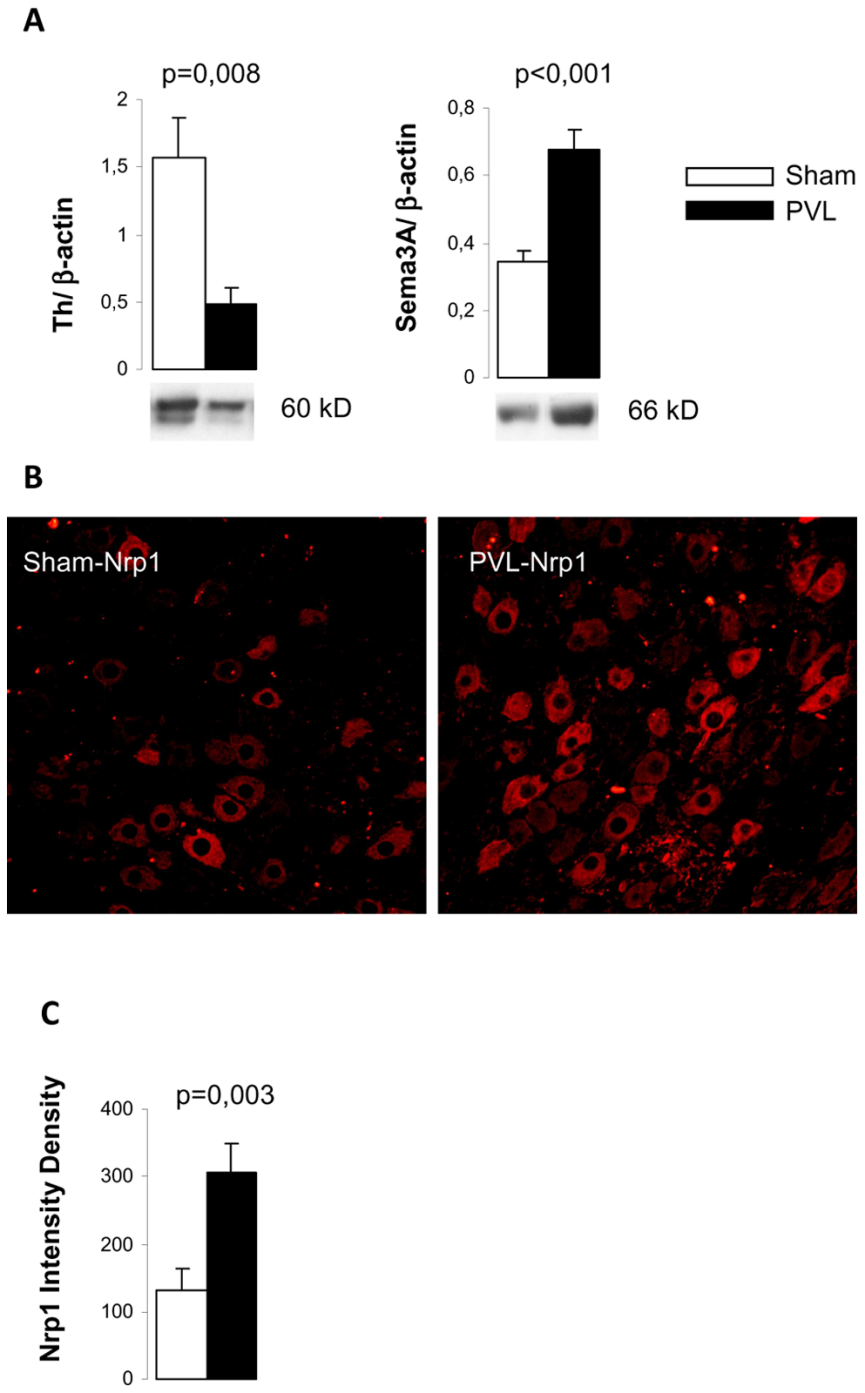
Tyrosine hydroxylase (Th), semaphorine 3A (Sema3A) and neuropilin (Nrp1) in the superior mesenteric ganglion (SMG). (A) Bar diagrams showing Th and Sema 3A quantitation by Western blot analysis in sham (n = 6) and PVL (n = 7). Representative Western blots are shown below. (B) Representative images of Nrp1 immunoreactivity in sections of SMG at 400× from sham (n = 6) and PVL (n = 5). (C) Quantitation of intensity density of Nrp1 fluorescence in each experimental group.

In order to confirm the adrenergic deficit previously found in mesenteric arteries of PVL rats [4], [6], Th expression was analyzed by Western blot in the SMG of PVL and sham rats. A significant 70% decreased in Th expression was found in PVL animals (p = 0.008) (figure 4A). The increased expression of Sema3A in the SMG of PVL, already demonstrated in a previous work [6], was confirmed by Westen blot analysis in PVL animals compared to sham (p<0,001) (figure 4A). Moreover, immunofluorescent analysis showed that Sema3A receptor Nrp1, located at the cytoplasm of adrenergic neurons of the SMG, was significantly overexpressed in SMG neurons of PVL animals compared to sham (p = 0.003) (fig 4B and C). Finally, immunodetection of p75^NTR^, Sort1 and TrkA demonstrated their presence in the cytoplasm of adrenergic neurons in the SMG (figure S1). Moreover, co-localization of p75 ^NTR^ and Sort1 was observed (figure S2).

### The axonal retraction/apoptotic pathways are activated in the superior mesenteric ganglion from PVL rats

**Figure 5:**
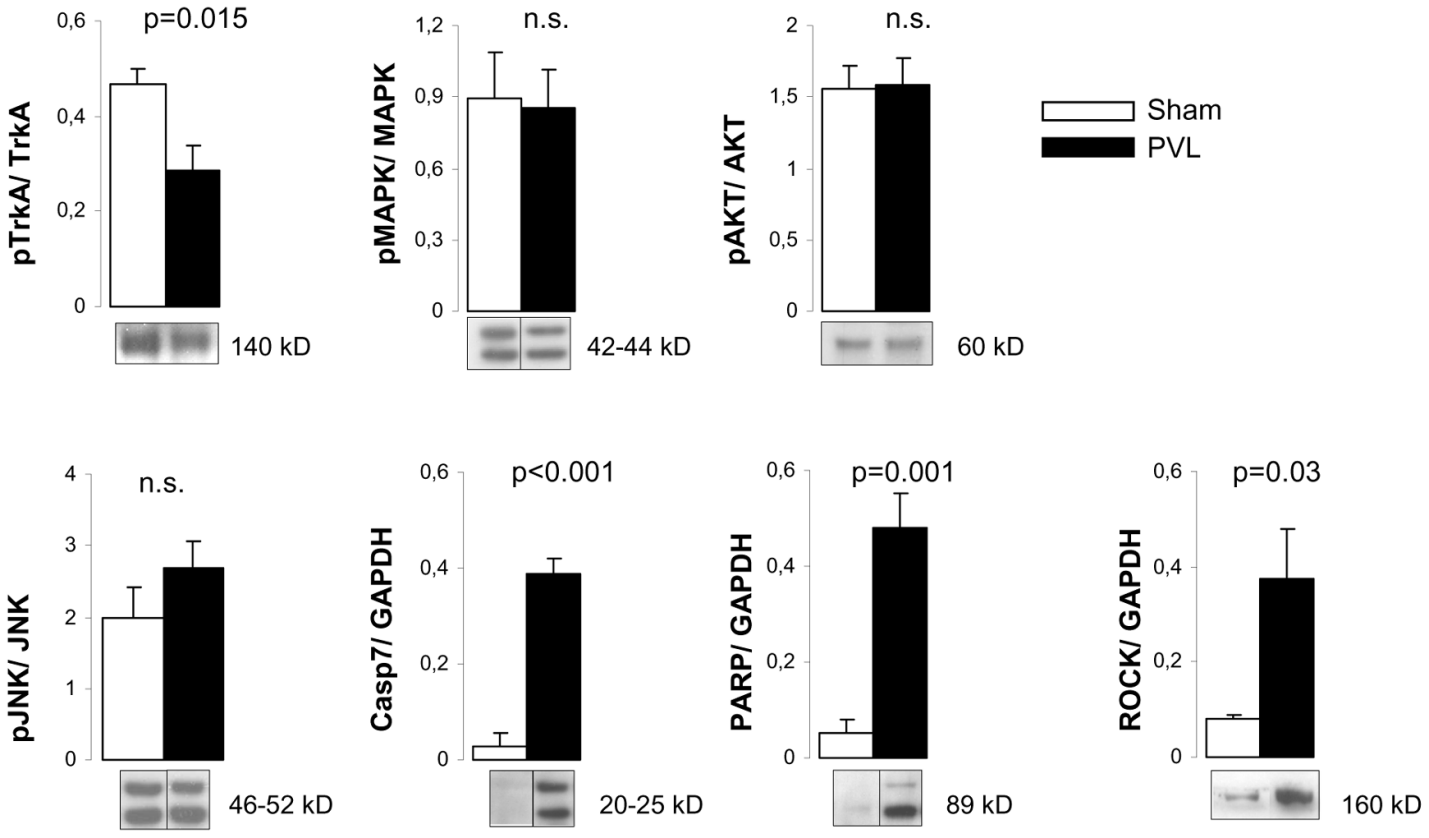
Expression of proteins involved in axonal survival and death pathways in the superior mesenteric ganglion. Bar diagrams showing quantitation by Western blot of (A) the ratio of phosphorylated and total forms of tyrosine kinase receptor A (TrkA), mitogen activated protein kinase (MAPK), protein kinase B (AKT) and (B) c-Jun N-terminal kinase (JNK), cleaved caspase 7 (Casp-7), poly(ADP-ribose)polymerase (PARP) and Rho kinase (ROCK), in sham (n = 7) and PVL (n = 7). Representative Western blots are shown below. Only bands corresponding to the phosphorylated form are exemplified in the case of the quantitation of ratios. Grouping of bands from different parts of the same gel are denoted by dividing lines.

Expressions of different proteins involved in the signaling pathways activated by either pro-NGF/NGF or Sema 3A were studied (figure 1). TrkA is the specific receptor for NGF and its activation leads to proliferative and prosurvival signaling through MAPK/AKT pathways. Although TrkA expression was not different between sham and PVL rats (not shown), its activated form, pTrkA, was decreased in PVL animals showing a decreased pTrkA/TrkA ratio in PVL rats compared to sham (p = 0.015) (figure 5). However, MAPK and AKT ratios showed no differences between PVL and sham groups (figures 1 and 5). In contrast, the apoptotic and axonal retraction pathways, activated by pro-NGF-p75^NTR^ and Sema3A-Nrp1, respectively, showed changes in PVL rats. On the one hand, the apoptotic cascade seemed to be activated in PVL compared to sham, demonstrated by strong increments in cleaved forms of Casp7 (25 and 20 kD) (p<0.001) and in the inactive form of PARP (p = 0.001). On the other hand, we were unable to find differences in JNK (not shown) or pJNK expression between PVL and sham rats (figure 5). Finally, our results demonstrated that ROCK expression, which regulates axonal retraction in response to Sema3A signaling, was largely increased (>3-fold) in PVL rats compared to sham (p = 0.03) (figures 1 and 5).

### Gambogic amide improves hemodynamic alterations and sympathetic atrophy in PVL rats

PVL animals treated with vehicle exhibited the characteristic hemodynamic changes observed in this model of portal hypertension (table 1). In contrast, PVL animals treated with gambogic amide (PVL-GA) improved hemodynamic parameters, except for PP, being significantly different from PVL vehicle. Although not achieving normal state sham values (shown in table 1 as a reference) PVL-GA animals presented a distinct intermediate hemodynamic state.

**Table 1:** Analysis of hemodynamic measurements of sham and PVL rats after gambogic amide or vehicle administration.

	PP (mmHg)	MAP (mmHg)	SMABF (ml/min.100 g)	SMAR (mmHg/ml.min.100 g
**Sham (n = 8)**	10,2±0,2	121,9±2,0	4,4±0,4	26,9±2,4
**PVL V (n = 8)**	15,7±0,4	97,4±2,7	7,2±0,5	11,7±0,9
**PVL GA (n = 8)**	14,6±0,8	111,6±4,6 *	5,8±0,39 *	16,3±0,6 **

p<0.05, ** p<0.001 compared with PVL V.

**Figure 6:**
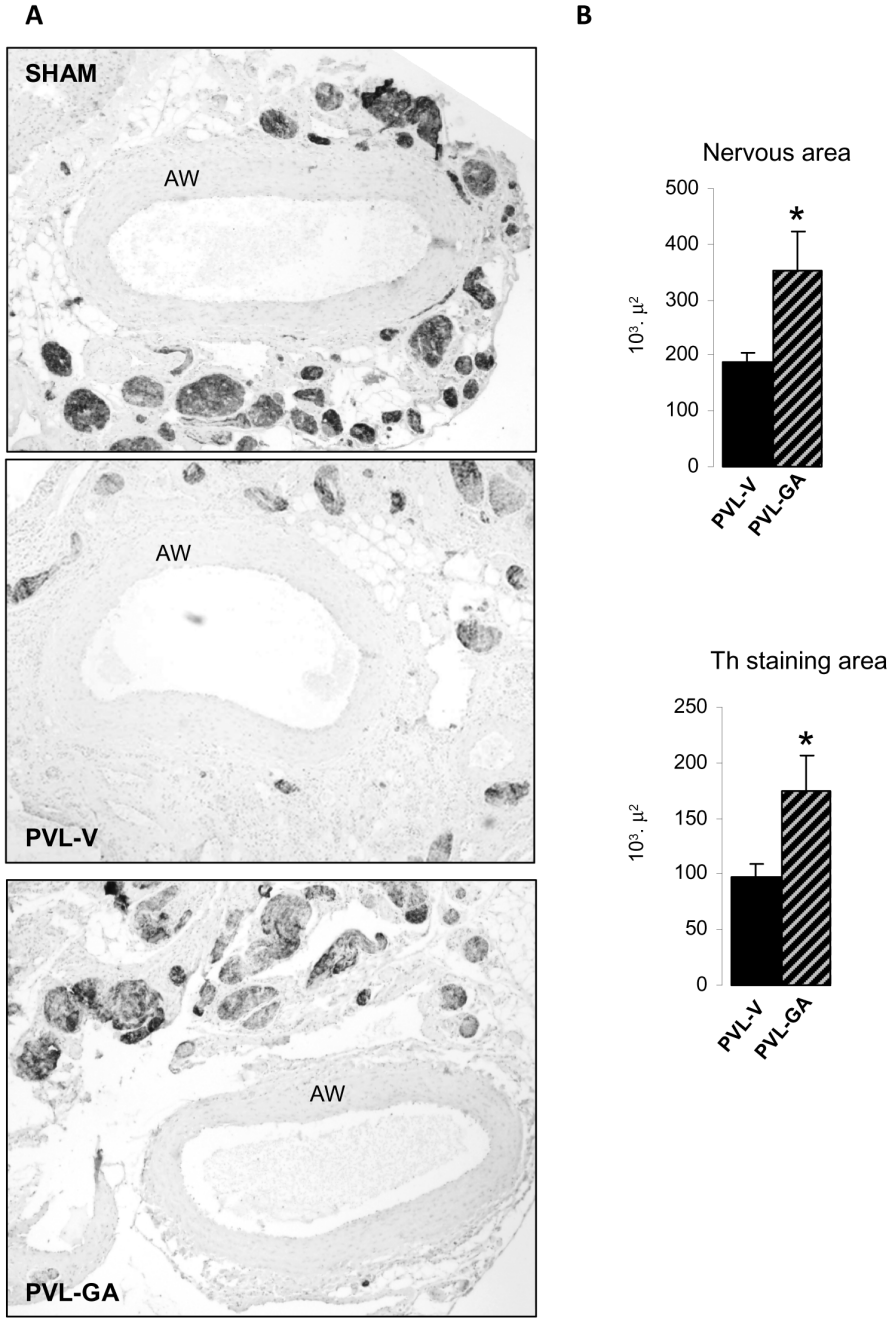
Analysis of sympathetic atrophy in the superior mesenteric artery (SMA) after gambogic amide administration. (A) Representative images of tyrosine hydroxylase (Th) immunostaining at 40× showing transversal sections of complete arterial wall (AW) surrounded by nervous structures from sham (showed as a normal state reference), PVL vehicle (PVL-V) (n = 6), PVL gambogic amide (PVL-GA) (n = 6), *p<0.05, **p<0.001, compared to PVL-V. (B) Bar diagrams showing immunohistochemical quantitation of total nervous area and Th staining area in nerves surrounding the SMA from experimental groups PVL-V and PVL-GA.

Analysis of the total nervous area surrounding the outer part of the arterial wall in SMA sections and the Th staining area within these nervous structures showed that both parameters were significantly lower in PVL vehicle than PVL-GA. Gambogic amide treated animals revealed less nerve atrophy demonstrated by a significant increase in total SMA nervous area (p = 0.03). Regarding the Th expressing area within the nervous structures PVL-GA animals also showed a significant increment compared to PVL vehicle (p = 0.04) (figure 6).

### Axonal regression/apoptosis within the superior mesenteric ganglia is decreased in PVL rats treated with gambogic amide

**Figure 7:**
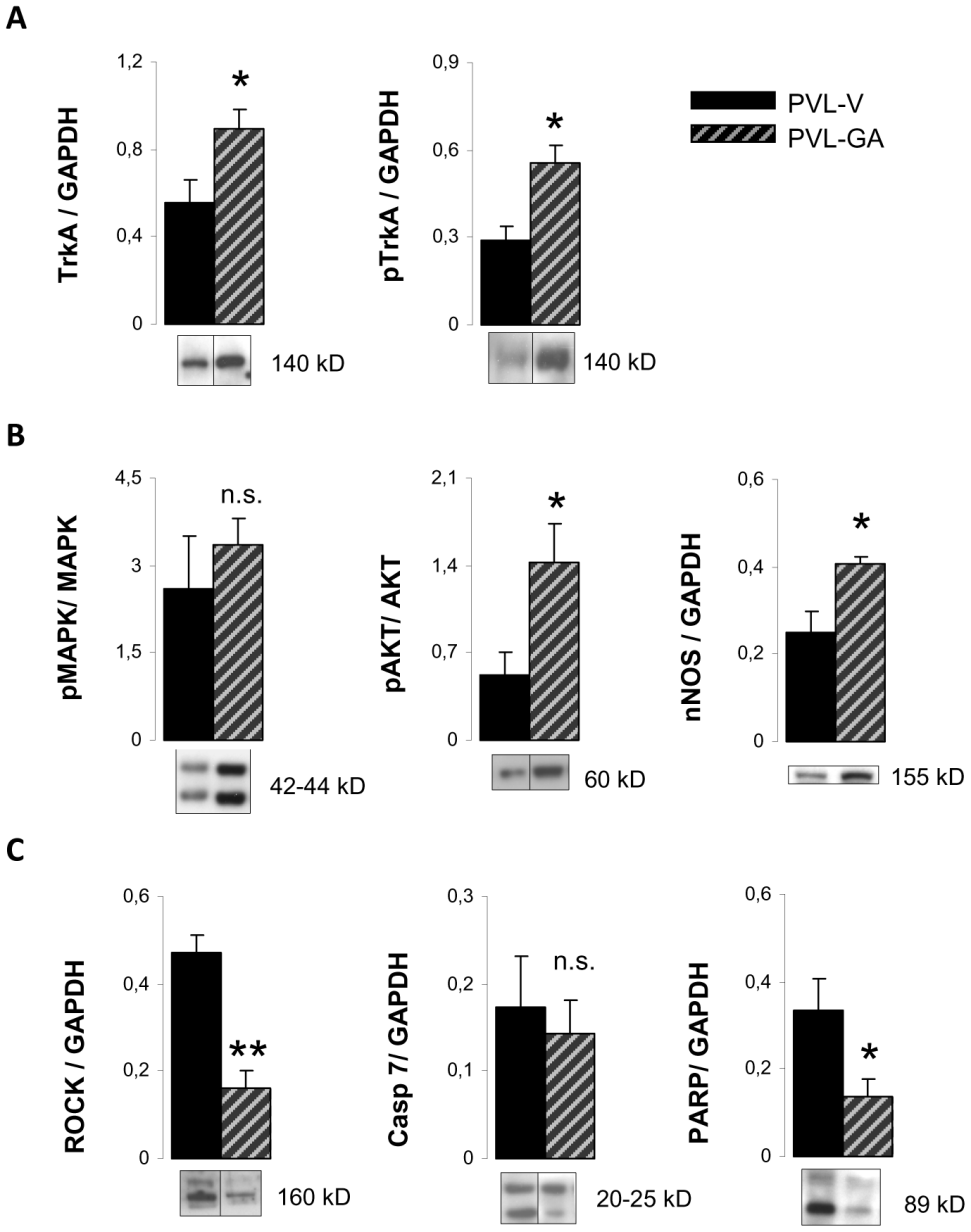
Protein expression in the superior mesenteric ganglion after gambogic amide administration. Bar diagrams showing quantitation by Western blot analysis of (A) tyrosine kinase receptor A (TrkA), phospho-TrkA (pTrkA), (B) the ratio of phosphorylated and total forms of mitogen activated protein kinase (MAPK) and protein kinase B (AKT) and neuronal nitric oxide synthase (nNOS), (C) Rho kinase (ROCK), cleaved caspase 7 (Casp-7) and poly(ADP-ribose)polymerase (PARP) in PVL treated with vehicle (PVL-V) (n = 8) or gambogic amide (PVL-GA) (n = 8). Representative Western blots are shown below. Only bands corresponding to the phosphorylated form are exemplified in the case of the quantitation of ratios. Grouping of bands from different parts of the same gel are denoted by dividing lines.

Administration of the TrkA agonist, gambogic amide, to PVL rats also changed the expression of most proteins implicated in neuronal growth/apoptosis pathways (figure 1). First of all, PVL-GA animals showed a significant increase in TrkA and pTrkA compared to PVL vehicle (p = 0.04 and p = 0,012, respectively). Consequently, pMAPK/MAPK and pAKT/AKT ratios were also significantly increased in PVL-GA compared to PVL vehicle animals (figure 7). This increased pAKT/AKT ratio suggested that it might be interesting to study the expression of nNOS, since it is the main source of NO in this tissue. As shown in figure 7, there was a significant 62% increase in nNOS expression in PVL-GA with respect to PVL vehicle rats (p = 0.008). On the other hand, compared with PVL vehicle, ROCK expression was significantly diminished in PVL-GA (p<0.001). Although cleaved Casp7 expression presented a slight non significant decrease in PVL-GA compared to PVL vehicle, the expression of cleaved PARP showed a significant decrease in PVL animals (p = 0,04) (figures 1 and 7).

## Discussion

Based on previous studies, we have hypothesized that the observed post-ganglionic sympathetic nerve regression in the splanchnic area would lead to neurotransmission inhibition and vasoconstriction impairment and constitute an additional contributory factor to arterial splanchnic vasodilation of portal hypertension [3], [4], [6]. This sympathetic nerve regression could be the result of efferent sympathetic signals synapsing at the SMG level. The origin of this neural response being an afferent signal, coming from portal or mesenteric pressure increments and reaching the brain stem cardiovascular nuclei through the vagus nerve [5], [7], [8].

The results of the present study support our hypothesis and demonstrate that an altered neuromodulator expression at the level of mesenteric sympathetic ganglia, leading to sympathetic axonal regression and apoptosis, is implicated in the development of splanchnic vasodilation. By pharmacological manipulation, the neuromodulation profile can be improved, the sympathetic atrophy ameliorated and the hemodynamic alteration of portal hypertension partially reverted.

### The neural signaling in portal hypertension

In this work, we have first analyzed two intermediate structures of this neural routing, the nodose ganglia in the afferent direction and the intermediolateral nucleus of the spinal cord in the efferent direction. The increased expression of the neuropeptide CGRP in the nodose ganglia of PVL rats, containing afferent capsaicin sensitive neurons, confirms an enhanced afferent signal through the vagus nerve in portal hypertension (figure S3). In this sense, it has been recently shown that capsaicin vagal denervation completely blocks this increased sensitive signal and abrogates hyperdynamic circulation in PVL rats [7]. In the efferent direction, VAChT immunostaining enabled localization of cell bodies of pre-ganglionic neurons at the intermediolateral nucleus of the spinal cord (segments from T9 to T13) that synapse later with post-ganglionic sympathetic neurons in the SMG. Furthermore, Sema3A positive immunostaining of these neurons supported the origin of Sema3A overexpression observed previously at the SMG of PVL in these cholinergic pre-ganglionic fibers [6]. Therefore, the efferent signal coming from the brain cardiovascular nuclei may induce the pre-ganglionic sympathetic neurons at the intermediolateral nucleus to produce Sema3A. From there, Sema3A could travel and be secreted at the SMG, affectting post-ganglionic adrenergic neurons and leading to axon growth inhibition, retraction or death [16]–[18] (figure S3). It is well known that the development of hyperdynamic splanchnic circulation in portal hypertensive rats is an angiogenesis-dependent process that can be markedly inhibited by blockade of the VEGF signaling pathway [19], [20]. The increase in Sema3A might also be necessary to redirect axons to innervate newly formed blood vessels from preexisting vasculature during portal hypertension [21]. In this process, activation of neuronal death or axonal retraction might be a collateral damage of the increased Sema3A levels.

### Altered neuromodulator expression in superior mesenteric ganglia

Data from the present study expands and completes our previous results [6] on the exploration of neuronal markers in the SMG and its signaling pathways in portal hypertension. First of all, we verified the adrenergic deficit previously observed in mesenteric arteries, in the SMG of PVL rats [3], [4], [6] through the analysis of Th expression, a well-accepted marker of sympathetic activity. Increased expression of Sema3A and its receptor Nrp1 were also confirmed in the SMG of PVL rats. Moreover, to rule out the possibility that Sema3A, NGF or proNGF could be acting on different cells other than adrenergic neurons of the SMG, we analyzed the expression of receptors Nrp1, TrkA, p75^NTR^ and Sort1 by immunofluorescence and found that they only localized in the cytoplasm of adrenergic neurons. These results reinforce the idea that an altered activity in the sympathetic neurons of the SMG is in the origin of mesenteric sympathetic atrophy.

TrkA and p75^NTR^ are mature NGF coreceptors, wherein TrkA transduces survival and differenciative signaling and p75^NTR^modulates the affinity and selectivity of TrkA activation (9) (figure 1). On the other hand, proNGF engages p75^NTR^ and sortilin to iniciate p75^NTR^ –dependent apoptotic cascade [22], [23] or to activate, in partnership with Sema3A, the signaling pathway leading to growth cone collapse and axonal retraction [15]. In turn, Sema3A is a negative regulator of NGF-induced neurite outgrowth via de-phosphorylation of TrkA, independently of its growth cone repulsion activity [12] (figure 1). Our data pointed out to the activation of p75^NTR^ inducing axonal regression and apoptosis and to the inactivation of TrkA by Sema3A. Although TrkA expression did not change in portal hypertensive animals, our results showed a diminished expression of its active phosphorylated form. This was predictable as the observed overexpression in Sema3A could be acting on TrkA de-phosphorylation of residues Tyr490 and Tyr785 (SCH and PLCγ docking sites, respectively) [12], [24]. Surprisingly, we were no able to find differences in the downstream signaling cascade regulated by these two tyrosine residues; levels of MAPK or its active phosphorylated form showed no difference between sham and PVL rats. We don’t have an exact explanation for this result but our *in vivo* tissue-type analysis cannot discriminate between the expression of MAPK originated only from NGF-TrkA stimulation in adrenergic neurons and from the many other sources of MAPK activation [25].

On the contrary, the signaling pathways activated by proNGF/Sema3A leading to neuronal apoptosis and axonal regression were markedly activated in PVL animals. Sema3A repulsive guidance signaling converges upon ROCK which propagates this signal down to the cytoskeleton acting as a key mediator of neuron growth inhibition and axon retraction [26] (figure 1). Moreover, caspases have been shown to irreversibly activate ROCK by truncation and generation of a constitutively active form [27]. Our results showed that the caspase cascade is activated in PVL rats demonstrated by important increments in cleaved Casp7 expression and in the inactive form of PARP; this caspase activation might be unrelated to the JNK pathway signaling. Simultaneously, the increased ROCK expression points out to an activated process of axon retraction in portal hypertension, either from Sema3A signaling or from caspase-dependent apoptosis.

### Treatment with gambogic amide

In the last part of this study, we tested the hypothesis that by manipulating this altered survival/death neuromodulator pathways, substantial changes in sympathetic atrophy and splanchnic vasodilation would be observed. We have tested gambogic amide, a small-molecule agonist for TrkA receptor, to reverse or modify TrkA altered expression or the intermediary molecules in its signaling pathway [28]. The selective interaction of gambogic amide with the cytoplasmic juxtamembrane domain of TrkA triggers the phosphorylation of TrkA at tyrosine residues Y490 and Y751, elicits AKT and MAPK activation and prevents neuronal cell death [28]. The results obtained from the analysis of proteins involved in growth and death neuronal pathways in the SMG agree with the observed improvement of the sympathetic atrophy in the nervous fibers surrounding the SMA of treated PVL rats. As a primary effect, gambogic amide significantly induces the activation of TrkA, consequently increasing AKT and MAPK expression above the expression levels found in PVL vehicles. This increase in neuronal growth and proliferation, by itself, could be counteracting the neurodegenerative process demonstrated in the SMG of PVL rats. Moreover, the dramatic decrease in ROCK and PARP content of PVL rats treated with gambogic amide, suggests that the activation of the AKT survival pathway promotes not only inhibition of axon retraction via ROCK, but also blockade of the caspase cascade leading to apoptosis. To this inhibition might also contribute the overexpressed nNOS by the generation of nitric oxide (figure 1) [29], [30].

Our results showed that the hemodynamic alteration and sympathetic atrophy are simultaneously improved in PVL rats treated with gambogic amide. We believe that the improvement in hemodynamic parameters is mostly due to the prevention of sympathetic atrophy. An increase in both sympathetic nervous area and tyrosine hydroxylase expression in PVL-GA probably contribute to restore a normal state of vasoconstriction. However, the hemodynamic improvement is not complete, because PVL-GA rats do not show the hemodynamic parameters of healthy individuals. The main reason for this incomplete improvement is that sympathetic atrophy only partially explains mesenteric vasodilation since other factors certainly intervene in this alteration. Another possible explanation to the lack of a complete hemodynamic/nervous restoration could come from the possible opposed effects of gambogic amide in different tissues in the mesenteric territory. Activation of TrkA in the SMG can promote the proliferation and survival of sympathetic nerve structures, followed by a consequent increment on adrenergic neurotransmission and eventually vasoconstriction. On the other hand, gambogic amide could be inducing at the same time SMA vasodilation via an increased phosphorylation of TrkA, AKT and eNOS.

In summary, during portal hypertension splanchnic arterial vasodilation is accompanied by sympathetic nerve atrophy. This sympathetic nerve regression is caused by a simultaneous inhibition of neuronal growth/survival pathways and the stimulation of neuronal apoptosis and axonal retraction processes, at the level of ganglionic sympathetic neurons. Reverting this altered neuromodulator alteration with a TrkA receptor agonist, partially prevents mesenteric sympathetic regression and improves the hemodynamic alteration of portal hypertension, indicating that sympathetic nerve regression is a contributory factor to splanchnic vasodilation in portal hypetension.

## Supporting Information

Figure S1 **Immunofluorescent detection of tyrosine kinase receptor A (TrkA), neurotrophin receptor p75 (p75NTR) and sortilin 1 (Sort1) in the sympathetic adrenergic neurones of the superior mesenteric ganglion.**
(TIF)

Figure S2 **Immunofluorescent co-localization of sortilin 1 (Sort1) and neurotrophin receptor p75 (p75NTR) in sympathetic adrenergic neurons of the superior mesenteric ganglion.**
(TIF)

Figure S3 **The neural pathway in portal hypertension.** NE: norepinephrine.
(TIF)

Data S1 Data Supplement
(DOC)
