# Supplementary figures and images for: Inhibition of Neuronal Apoptosis and Axonal Regression Ameliorates Sympathetic Atrophy and Hemodynamic Alterations in Portal Hypertensive Rats
Source: PLoS One. 2014 Jan 6;9(1):e84374. doi: 10.1371/journal.pone.0084374 (PMC3882227; doi:10.1371/journal.pone.0084374)

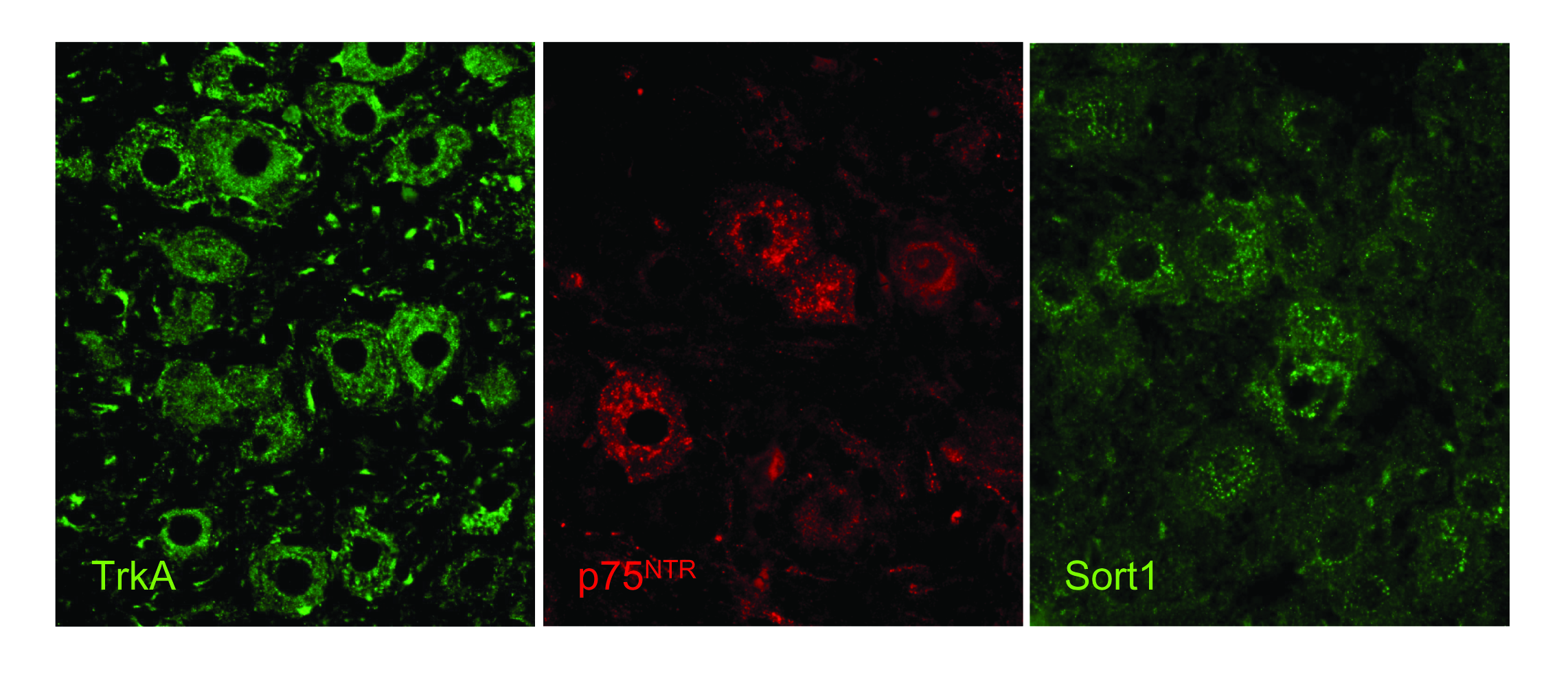

Supplement: Figure S1 — Immunofluorescent detection of tyrosine kinase receptor A (TrkA), neurotrophin receptor p75 (p75NTR) and sortilin 1 (Sort1) in the sympathetic adrenergic neurones of the superior mesenteric ganglion. (TIF) [file pone.0084374.s001.tif]

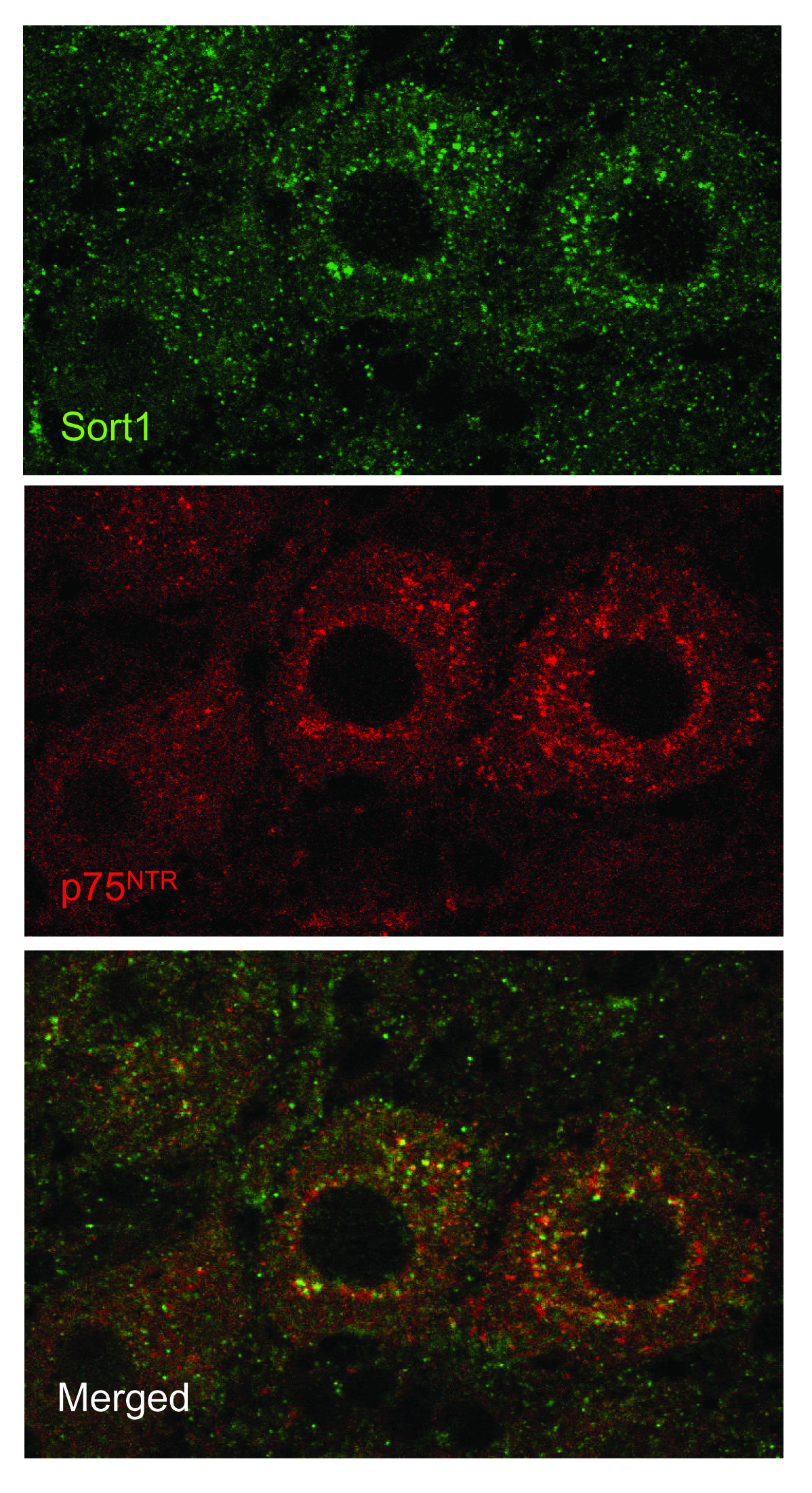

Supplement: Figure S2 — Immunofluorescent co-localization of sortilin 1 (Sort1) and neurotrophin receptor p75 (p75NTR) in sympathetic adrenergic neurons of the superior mesenteric ganglion. (TIF) [file pone.0084374.s002.tif]

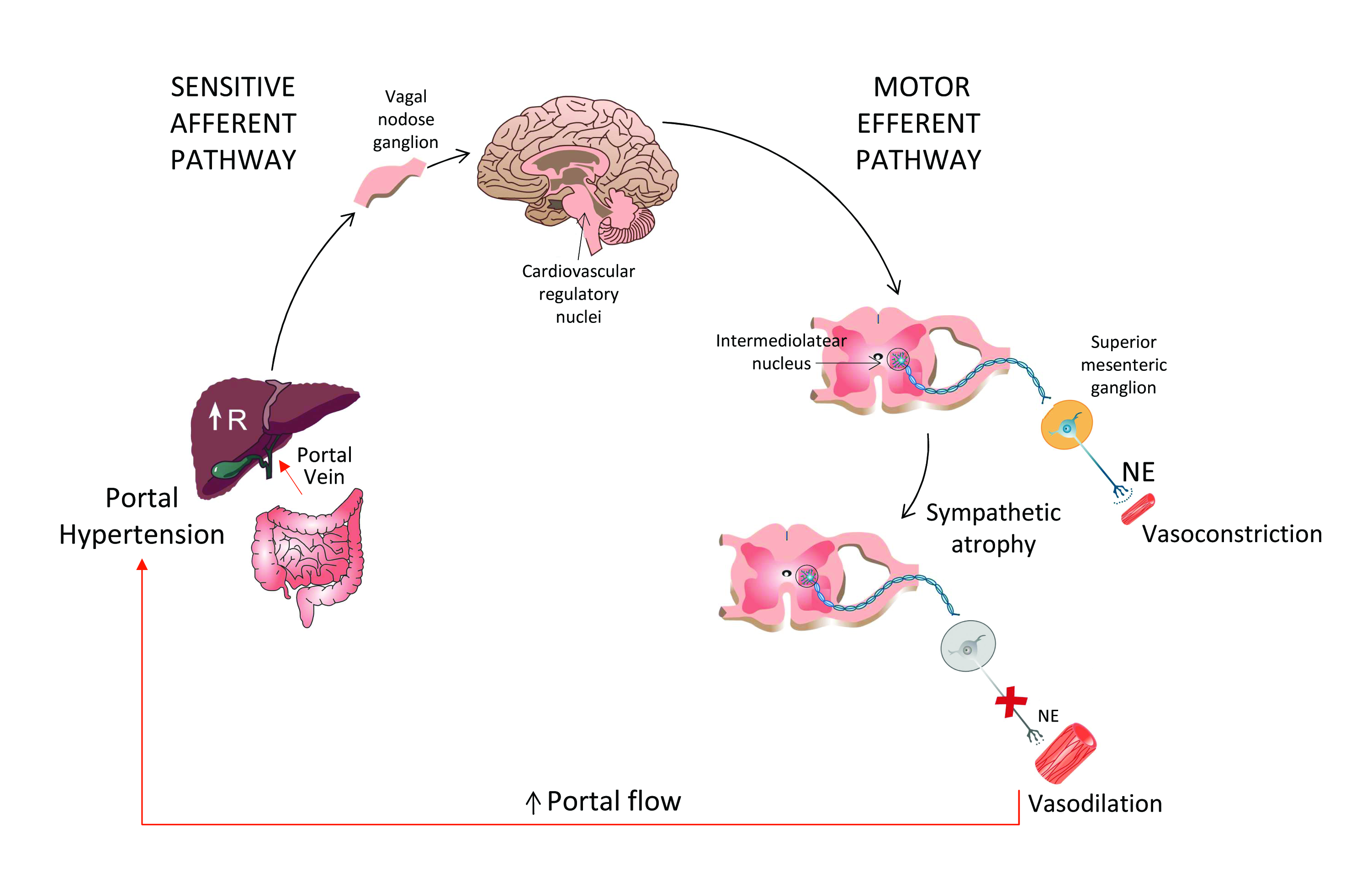

Supplement: Figure S3 — The neural pathway in portal hypertension. NE: norepinephrine. (TIF) [file pone.0084374.s003.tif]
