# Supplementary material for: Inhibition of Neuronal Apoptosis and Axonal Regression Ameliorates Sympathetic Atrophy and Hemodynamic Alterations in Portal Hypertensive Rats
Source: PLoS One. 2014 Jan 6;9(1):e84374. doi: 10.1371/journal.pone.0084374 (PMC3882227; doi:10.1371/journal.pone.0084374)
Supplement: Data S1 — Data Supplement (DOC) [file pone.0084374.s004.doc]

**DATA S1**

**Sample extraction**

The superior mesenteric ganglion located at the confluence of the superior mesenteric artery and the aorta, was visualized with a microscope Carl Zeiss OPML 9FC, dissected free of surrounding tissue and removed by cutting the nerve fibers with scissors. Superior mesenteric ganglia were placed either in formalin and 50% ethanol before paraffin treatment, or in liquid nitrogen and kept at -80ºC until processed. Superior mesenteric arteries, harvested from the aortic origin to the initiation of ramifications, were kept in 4% formalin for approximately 20 hours and changed to a 50% ethanol solution before paraffin treatment. Nodose ganglion samples were separated from the internal carotid artery and the internal jugular vein, with the aid of a microscope (Carl Zeiss OPML 9FC, Germany), immersed in liquid nitrogen and stored at -80° C. Finally, in order to extract spinal cord samples, once animals were dead, a portion of the spine (from T9 until T13) was extracted (vertebrae plus spinal cord). After 48 hours in 4% paraformaldehyde at 4ºC, the spinal cords were separated from vertebrae and immersed in 30% sucrose in 0.1mol/l phosphate buffer during 1-3 days at 4ºC, frozen in methyl butane and kept at -80ºC.

**Western blot analysis**

Tissue samples were homogenized in 250 ml RIPA buffer and 15 ml of a protease inhibitor cocktail (Sigma-Aldrich, St Louis, MO, USA). Protein quantification in the supernatants was performed by the BCA assay (Pierce, Rockford, IL, USA). Equal amounts of protein extracts (4-20μg, depending on the analyzed protein) were separated on 10–12% sodium dodecyl sulphate polyacrylamide gel electrophoresis (depending on the protein size), blotted onto polyvinylidene difluoride membranes (Invitrogen, Carlsbad, CA, USA) and incubated with primary antibodies. Bands were quantified by the QUANTITY ONE software (Bio-Rad Laboratories, Hercules, CA, USA).

**Immunofluorescence**

Spinal cord sections were cut at 8 μm using a cryostat (Leica CM3050S). Slides were blocked in a 1:20 dilution of goat serum for 1 h at RT and incubated with primary antibodies at 4ºC during 48 h. Bound antibodies were incubated 1 h at RT with anti-goat IgG DyLight 594 (diluted 1:500) (Bethyl, Montgomery, TX, USA) for VAChT and with anti-rabbit-FITC (diluted 1/500) (Abcam, Cambridge, UK) for Sema3A. Paraffin-embedded sections of SMG were blocked for 1 h and incubated overnight at 4ºC with primary antibody and then1 h at RT with anti-rabbit-FITC. Slides were mounted in Vectashield hard set mounting medium for fluorescence and visualized with an optical microscope Olympus BX61 (Olympus, Hamburg, Germany). Quantitative analysis of Nrp-1, was performed with the Image J analysis software (<http://rsbweb.nih.gov/ij/>), as previously described [6].
